# Parents’ and healthcare professionals’ experiences with the content of an individual care plan for pediatric palliative care: a mixed-method study

**DOI:** 10.1177/26323524241277572

**Published:** 2024-09-18

**Authors:** Chantal Y. Joren, Marijke C. Kars, Leontien C. M. Kremer, Suzanne C. Hofman, Hester Rippen-Wagner, Ria Slingerland-Blom, Chantal van der Velden, Meggi Schuiling-Otten, A. A. Eduard Verhagen, Judith L. Aris-Meijer

**Affiliations:** University of Groningen, University Medical Center Groningen, Beatrix Children’s Hospital, Hanzeplein 1, Groningen 9713 GZ, The Netherlands; Center of Expertise in Palliative Care Utrecht, Julius Center of Health and Primary Care, UMC Utrecht, Utrecht, The Netherlands; Princess Maxima Centre for Pediatric Oncology, Utrecht, The Netherlands; University Medical Center Utrecht, Wilhelmina Children’s Hospital, Utrecht, The Netherlands; Dutch Foundation Child and Hospital, Utrecht, The Netherlands; Dutch Centre of Expertise in Children’s Palliative Care, Utrecht, The Netherlands; Dutch Centre of Expertise in Children’s Palliative Care, Utrecht, The Netherlands; Dutch Centre of Expertise in Children’s Palliative Care, Utrecht, The Netherlands; University of Groningen, University Medical Center Groningen, Beatrix Children’s Hospital, Groningen, The Netherlands; University of Groningen, University Medical Center Groningen, Beatrix Children’s Hospital, Groningen, The Netherlands

**Keywords:** Individual Care Plan, pediatric advance care planning, pediatric palliative care

## Abstract

**Background:**

The Individual Care Plan (ICP) for pediatric palliative care translates the general guideline recommendations into a personalized plan for the child. Various documents exist in pediatrics globally, aimed at facilitating anticipatory care or coordinating end-of-life care. The ICP aims both, but user experiences have not been studied post-development.

**Objective:**

The overall aim is to enhance knowledge and understanding of the content of the ICP from the perspectives of parents and healthcare professionals (HCPs).

**Design and method:**

We conducted a mixed-method study using a convergent parallel design consisting of questionnaires and individual and focus group interviews among parents and HCPs having user experience with the ICP. The questionnaire and interview data were analyzed separately. Quantitative data were descriptively analyzed using mean, ±SD, and median. Qualitative data were thematically analyzed. A narrative approach and joint display were used to describe the results.

**Results:**

In total, 27 parents and 161 HCPs participated. Overall, the content of the ICP was seen as important and complete, but changes and additions were called for on language, structure, and content. The chapter on the needs and wishes of child and parents was considered most important. HCPs would like to see this chapter expanded to incorporate more advance care planning outcomes, and parents wished for this chapter to reflect better who their child is. HCPs mentioned missing a chapter for palliative sedation, mainly to guide other HCPs. The ICP was appraised as not user-friendly and might possibly improve by making the ICP available in a secure digital environment.

**Conclusion:**

To meet the needs of parents and HCPs considering importance and completeness of the content of the ICP and its user-friendliness, changes are necessary in the content of the ICP, and preferably the ICP should be made digitally available. Although various documents exist globally to facilitate anticipatory care or coordinating end-of-life care, it appears that the combination of describing the values and preferences of the child and parents, along with medical decisions and life-sustaining treatments, makes the ICP a unique and comprehensive care plan.

## Introduction

Globally, various documents exist aimed at facilitating anticipatory care and coordinating end-of-life care,^1–5^ such as advance directives and advance/shared care plans. However, there is limited insight into the exact content of these documents.^6^ In studies that do mention the content, a wide variation is found. In some documents the emphasis is placed on medical decisions and life-sustaining treatments,^1,2^ while in others the focus is on advance care planning by exploring the values and preferences of the child and family.^3–5^

The Individual Care Plan (ICP) 1.0 for pediatric palliative care (PPC) was developed to provide person-centered care for each individual child with a life-limiting or life-threatening condition.^7^ The ICP is based on the translation of the general recommendations of the Dutch guideline “palliative care for children” into a personalized plan.^8^ By means of the ICP, preferences, desires, and agreements regarding current and future care and treatment of the child can be documented and shared with involved healthcare professionals (HCPs) across lines of care, aiding coordination, continuity, and quality of care for the individual child. The ICP 1.0 is available as a Word form consisting of 10 chapters, including chapters on general information, social and psychological aspects, needs, wishes, and goals, medication, pharmacological and non-pharmacological symptom management, and nutrition, see Supplement Material 1.^7^

Although several studies reveal that documents similar to the ICP are beneficial in PPC,^6,9,10^ it remains unclear what the essential content of an ICP for PPC is. From practice we know that the ICP is not used for all children with life-limiting or life-threatening disease and that parents and HCPs find the ICP difficult to work with. However, we do not exactly know what this means, as no previous studies have investigated user experiences. Therefore, the aim of this study is to enhance knowledge and understanding of the content of the ICP from the perspective of parents and HCPs. The following research questions are answered: (1) the importance, completeness, and user-friendliness of the content of the ICP according to parents and HCPs and (2) their experiences with the content of the ICP in daily practice.

## Methods

### Design

This study is part of a larger multi-phase mixed-method study in the Netherlands, which took place from 2021 to 2023. An extensive description of the study design was previously published elsewhere.^11^ This mixed-method cross-sectional study has a convergent parallel design,^12^ using questionnaires for parents and HCPs, individual interviews with parents and focus group interviews with HCPs. Data collection took place from April to August 2021. This design was used to leverage both strengths of quantitative research, objective measures from a larger sample size, and qualitative research, in depth data, so as to attain a more comprehensive understanding of parents and HCPs experiences with the content of the ICP.^12^ The reporting of this study conforms to the MMR-RHS checklist,^13^ see Supplemental Material for the completed form.

### Sample

We used criterion sampling for all respondents in both the quantitative and qualitative studies.^14^ Eligible participants were parents and bereaved parents of children for whom an ICP 1.0 was drawn up in the past 3 years and HCPs who work with children who receive PPC and who used the ICP 1.0 in a PPC setting.^11^

### Recruitment

#### Recruitment parents

All eight specialized multidisciplinary Children’s Palliative Care teams and all Children’s Palliative Care networks in the Netherlands were involved in the recruitment of participants.^11^ Children’s Palliative Care teams sent out email-invitations to parents for whose children an ICP had been drawn up. Parents could enroll themselves in the study, for either the questionnaire or the individual interview, or both. A call for participation, including an URL to the questionnaire, was posted on the social media canals of the Dutch Foundation Child and Hospital and Dutch Centre of Expertise in Children’s Palliative Care.

#### Recruitment HCPs

Children’s Palliative Care teams sent out invitation emails including the URL to the online questionnaire to all HCPs involved in ICPs, along with a call to invite other HCPs within their network. Children’s Palliative Care networks sent out similar invitation emails to HCPs involved in the research project. Furthermore, a call for participation was posted on the social media channels of the Dutch Foundation Child and Hospital and Dutch Centre of Expertise in Children’s Palliative Care.

For the focus group interviews with HCPs, the Children’s Palliative Care teams and networks identified all HCPs who had experience with the ICP 1.0. Invitations were sent to the selected HCPs from those who were identified. Variation was sought with respect to regions, functions, lines of care, and disciplines. HCPs could enroll themselves for the focus group interviews through the project website.

**Figure 1.**
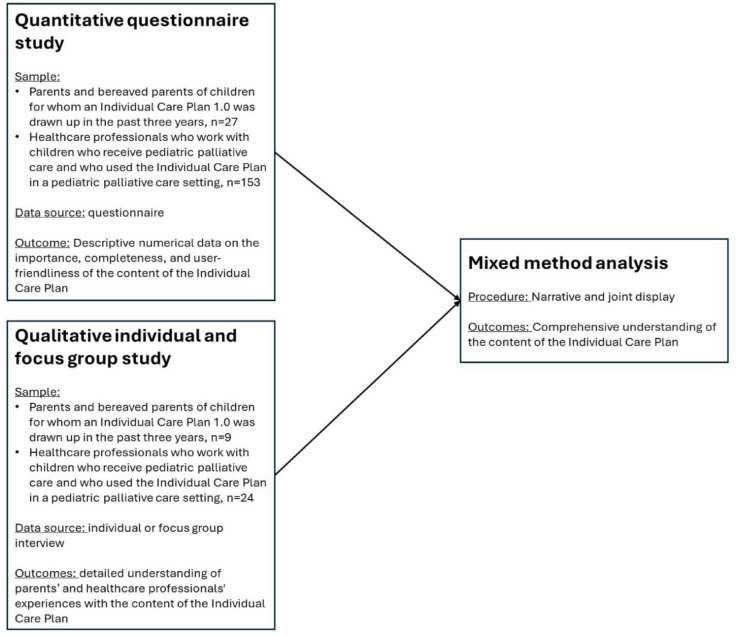
Mixed-method convergent parallel design.

### Data collection and measurements

#### Online Questionnaires

The questions from the ICP 1.0 pilot study,^7^ in which the ICP was developed, were used as foundation for the questionnaires. The questionnaires were extended with study-specific questions on background variables, content, process, and structure of the ICP 1.0. Content was operationalized using importance, completeness, and user-friendliness. For each chapter of the ICP parents and HCPs were asked how (1) important, (2) complete, and (3) how user-friendly they found the chapter on a Likert scale from 0 = completely disagree to 4 = totally agree. Even though some organizations used an adapted version of the ICP 1.0, the questions regarding importance, completeness, and user-friendliness in the questionnaire focused on the use of the original ICP 1.0. However, participants were asked if they used an adapted version.

#### Individual and focus group interviews

The digital semi-structured individual and focus group interviews explored the experiences of parents and HCPs with ICP 1.0. The interviews were moderated by JA (female, postdoc researcher), who was trained in conducting interviews, using the platform Teams. MK (former pediatric nurse, senior qualitative researcher) was present as observer at two of the focus groups. Topic guides were designed based on literature and expertise from the research team for parents, HCPs with experience with working with the ICP 1.0, and HCPs experienced with drawing up the ICP 1.0, see Supplemental Materials 2 and 3. The topic guide for the focus group interviews was tested by interviewing two HCPs. Four groups of maximum six HCPs were interviewed, each lasted 1.5 h. Two focus groups included HCPs with experience with working with the ICP 1.0, and two with HCPs experienced with drawing up the ICP 1.0. This distinction was made to strengthen group dynamics, as not all questions would be relevant to both groups of HCPs. The individual interviews with parents lasted approximately 1 h.

### Data analysis

#### Quantitative

The quantitative data were descriptively analyzed in SPSS version 28 using mean, ±SD, and median. In addition, Fisher Exact tests were used to analyze for differences between HCPs groups. A *p* value of 0.05 was used to indicate statistical significance.

#### Qualitative

All interviews were videotaped, transcribed verbatim, and pseudonymized. Videotapes were deleted after transcription. Data were thematically analyzed using open and axial coding.^15^ To get familiar with the data, two individual interviews and one focus group interview were independently read and coded by CJ, MK, and JA. Together, the codes and interpretations of the fragments were compared and discussed till consensus was reached, resulting in an adapted code tree. Based on the code tree, all transcripts were coded by CJ. The code tree was evaluated with the team and adjusted during the process. After coding all interviews, the team identified key themes and related subthemes. The analysis was supported by Atlas.ti 9.

#### Mixed-method integration and analysis

In line with a convergent parallel design, the quantitative and qualitative data were analyzed separately. After analysis, the results were integrated in a joint display and compared to assess confirmation, expansion, or discordance.^16^ Confirmation occurred if the findings from the questionnaire and interview confirmed the results of the other.^16,17^ Expansion occurred if the findings from the two types of data diverged and expanded insight of the content of the ICP. Discordance occurred if the two types of data were inconsistent, contradictory, or disagreed with each other.^16,17^ For reporting the results, a narrative approach was used by describing the two types of data together on a theme-by-theme basis.^16^

## Results

### Respondents

In total, 27 parents participated in the questionnaire, of whom 9 participated in both the interview and questionnaire (Table 1). A total of 161 HCPs participated, of whom 153 in the questionnaire and 24 in the focus group interviews. Of those, 16 HCPs participated in both the questionnaire and focus group interview (Table 2).

**Table 1.** Characteristics of the respondent group parents.

Characteristics	*n* *=* 27^a,b^ (*n*, %)
Age, mean (min–max)	44 (26–60)
Highest completed education	
Basic vocational education and training (EQF^c^ = 2)	3 (11.1)
Secondary vocational qualification (EQF^c^ = 4)	10 (37.0)
Higher general secondary education/pre-university education (EQF^c^ = 4)	2 (7.4)
Bachelor’s degree (EQF^c^ = 6)	12 (44.4)
Child’s main diagnosis	
Cardiological	2 (7.7)
Metabolic	4 (15.4)
Neurological	8 (30.8)
Oncological	12 (46.2)
Child alive/child deceased	
Alive	6 (25.9)
Deceased	20 (74.1)
Age child at inclusion, mean (min–max)	10 (0–25)
Age at death, mean (min–max)	11 (0–19)

a All parents who were interviewed also completed the questionnaire.

b Sometimes the total *n* = 26 instead of *n* = 27, due to participation of both parents in the questionnaire.

c European Qualifications Framework.

**Table 2.** Characteristics of the respondent group healthcare professionals.

Characteristics	Total (*n* = 161)	Questionnaire^a^ (*n* = 153)	Focus groups^b^ (*n* = 24)
Gender female/male	148/13	140/13	22/2
Age, mean (min–max)	NA	45 (23–64)	NA
Children’s Palliative Care network^c^			
Northeast	33	30	6
Southeast	14	13	2
Limburg/Brabant	19	19	4
Southwest	9	8	2
Holland-Rijnland	21	20	3
North-Holland and Flevoland	19	18	2
Utrecht	26	25	5
Network unknown	20	20	—
Profession			
Medical doctor	32	27	7
General practitioner	12	12	0
Nurse	90	87	16
Other	27	27	1
Setting			
Hospital	63	56	14
Primary care	98	97	10
Role ICP			
Draw up ICP	63	58	8
Working with, did not draw up ICP	97	95	8
Both	NA^c,d^	NA^c,d^	8

a This also includes healthcare professionals who participated in the focus groups (*n* = 16).

b This also includes healthcare professionals who have completed the questionnaire (*n* = 16).

c The seven Children’s Palliative Care networks in the Netherlands.

d In the questionnaire respondents were asked what role they generally have regarding the ICP.

ICP, Individual Care Plan; NA, not available.

### Findings

First, the quantitative results will be presented regarding the importance and completeness of the content of the ICP 1.0 and the user-friendliness of the ICP 1.0. Second, the qualitative results regarding the importance, completeness, and user-friendliness of the ICP 1.0 will be presented separately. Table 3 shows the joint display of quantitative, qualitative, and mixed-method findings.

**Table 3.** Joint display of quantitative, qualitative, and mixed-methods findings.

Major topics	Quantitative results			Qualitative results		Mixed-method comparison
		Parents	HCPs	Parents	HCPs	
		Mean (±SD)	Mean (±SD)			
Importance	Overall* 1. 2. 3. 4. 5. 6. 7. 8. 9. 10.	3.42 (0.43) 3.11 (0.75) 3.50 (0.58) 3.32 (0.63) 3.62 (0.50) 3.35 (0.63) 3.56 (0.58) 3.50 (0.76) 3.04 (0.75) 3.42 (0.58) 3.04 (0.87)	3.28 (0.52) 3.30 (0.68) 3.55 (0.64) 3.44 (0.69) 3.58 (0.62) 3.42 (0.80) 3.30 (0.67) 3.46 (0.67) 2.91 (0.89) 3.11 (0.87) 2.83 (0.83)	“Personally, I think things like that are the most important, because the rest goes a long way if you just have a good nursing team. [. . .] While that actually hardly came to the fore.” (# 8) “Yes, how you communicate with [child]. How do you do that. . . almost all disabled children cannot speak and every child communicates differently [traceable information]. And yes, how can you best communicate with such a child?” (#2)	“I think that as soon as the diagnostics are somewhat clear, the ACP conversation should start. And if you start those ACP conversations, you will eventually end up with an ICP, because if you write down conclusions and form a policy, you will end up with an ICP. So, I think that ACP should play a very important role in this and that it cannot be viewed separately from an ICP.” (Nurse, primary care, working with ICP, #10). “If there is room, I discuss the expected scenarios with my patients or their parents. And I discuss that because I have already asked where that fear lies. What do you like? What do you want, what don’t you want? What that could look like. So I think those needs and wants are the most important.” (Pediatrician, working with ICP, #1) “[. . .] the integrated collaboration, that’s really crucial. Indeed, having an overview of: these are the contacts, here you can reach out. Yes, that’s just very nice to have.” (Nurse, primary care, working with ICP, #10)	*Expansion* Voicing and knowing the parents needs and wishes explains the highest score of importance of chapter 4. *Expansion* The need to know which healthcare professionals are involved explains the high score for importance of chapter 2.
Completeness	Overall* 1. 2. 3. 4. 5. 6. 7. 8. 9. 10.	3.05 (0.69) 3.00 (0.87) 3.19 (0.94) 3.04 (0.92) 3.23 (0.82) 2.96 (0.93) 3.20 (0.76) 3.08 (0.93) 2.77 (0.82) 3.08 (0.84) 3.04 (0.77)	2.97 (0.47) 2.98 (0.66) 3.06 (0.83) 2.98 (0.84) 2.96 (0.91) 2.93 (0.91) 3.00 (0.81) 3.06 (0.80) 2.79 (0.72) 3.02 (0.70) 2.77 (0.79)	“No, I think the document is actually quite complete” (#4) “The end of life part seems very short here. These were important parts for us.” (Questionnaire, chapter 4, open answer) “Normally you would think: oh dear, how sad that [the child] feels that. And when you read that [information on cessation of feeding], you think about it completely differently. That it was actually more relaxing for [the child] and that [the child] intestines were a bit calmer. [. . .]. So basically we were increasing suffering there. Yeah, so if you read that booklet, it actually gave us a lot of information. Without that booklet. . . . Well. I actually think that should be an addition.” (#3)	“I also really miss the palliative sedation, even though that is. . . Because that is of course true for those general practitioners. . . You know, they think: yes, it is good that there is a plan [ICP], but what should I do when the time comes [end-of-life]?” (Nurse, hospital, drawing up ICP, #2) “We sometimes miss when children come home, for example. . ., then they get a high and low bed, medication is supplied by their own pharmacy or done by another pharmacy, catheter care from that supplier. I would like it if, for example, you had a list in the ICP of: what was ordered where, from which supplier or so on. That you just have an overview of that.” (Nurse, home care, working with ICP, #8) “I think the most important thing is that it is complete, but that is also a bit of a pitfall effect, I think, because it is so complete, that it is very extensive.” (Nurse, home care, working with ICP #19)	*Expansion* Described as complete, but at the same time points for improvement are mentioned explaining the mixed scores for completeness of the chapters. *Discordance* Completeness is also seen as a pitfall which is contradictory to the scores for completeness.
User-friendliness	Overall* 1. 2. 3. 4. 5. 6. 7. 8. 9. 10.	3.02 (0.69) 3.00 (0.85) 3.23 (0.86) 3.00 (0.85) 3.08 (0.89) 2.89 (0.99) 3.31 (0.62) 2.69 (1.12) 2.77 (0.91) 3.15 (0.83) 3.12 (0.77)	2.79 (0.63) 2.77 (0.86) 2.85 (0.98) 2.89 (0.88) 2.85 (0.91) 2.65 (1.04) 2.98 (0.81) 2.45 (1.16) 2.71 (0.81) 2.86 (0.88) 2.82 (0.76)	“But digitally it might be much easier. Because I think that if you look at the fact that this plan is used for a care-intensive child, there is so much constant change, not only in terms of care, but also perhaps in health, the medical situation itself, that it is almost impossible to keep it up-to-date.” (#5) “Maybe use less difficult words.” (Questionnaire, chapter 7, open answer). “Comes across too businesslike.” (Questionnaire, chapter 1, open answer)	[. . .] and often in an acute situation or if the child suddenly deteriorates rapidly, you just want to have the most important things up front. And then I notice that it’s a matter of really searching in the file, so to speak, in the pages. (Nurse, primary care, working with ICP, #8) “And I really don’t have time to evaluate those schedules every time. For example, I already know that I have a child, a baby, and it is already two kilos heavier. Yes, that whole schedule no longer makes sense, but I really don’t have the courage to adjust the whole thing again. And you know, that was sent to home care and to the general practitioner and to the parents. And if you change it, you run the risk that you have to send a new version and that they use the old version. So that is still a struggle for me.” (Pediatrician, hospital, drawing up ICP, #13). “But I have also wondered how to make such a cumbersome plan manageable without sacrificing certain items. Because what is truly important? As mentioned earlier, for example, in the symptomatology, you look at it and think, well, what is applicable to this child?” (Nurse, hospital, drawing up ICP, #15).	*Expansion* The cumbersomeness of the plan and the wish for digitalization explains the lower user-friendliness scores.

Chapters of the Individual care Plan: 1. Care plan information; 2. General information; 3. Social map/psychosocial aspects; 4. Needs, wishes, and goals; 5. Medication including dosage; 6. Nutrition; 7. Symptomatology; 8. Alternative therapies and relaxation/wellness; 9. History of change; 10. Other. For each chapter within the Individual Care Plan the respondents could indicate to what extent they found the chapter important, complete, and user-friendly on a Likert scale from 0 = completely disagree to 4 = totally agree.

* The mean has been calculated for those respondents who answered all 10 questions on the importance, completeness, and user-friendliness.

HCP, healthcare professional; ICP, Individual Care Plan.

### Quantitative

Parents and HCPs were positive regarding the importance and completeness of the content of the ICP 1.0, and the user-friendliness of the ICP 1.0. Overall, parents were slightly more positive compared to HCPs regarding the importance, completeness, and user-friendliness of the ICP 1.0 in general and per chapter.

Both parents and HCPs scored chapter 4 “Needs, wishes, and goals” of the ICP as most important (parents: 3.62, HCPs: 3.58). Chapters 8 “Alternative therapies and relaxation/wellness’ (parents: 3.04, HCPs: 2.91), and 10 “Other” (parents: 3.04, HCPs: 2.83) were considered as least important. Chapter 6 “Nutrition” was scored as most complete (3.20) and user-friendly (3.31) by parents, and as most user-friendly by HCPs (2.98). Chapter 7 “Symptomatology” was seen as most complete (3.06) by HCPs. However, it was also indicated as least user-friendly by both parents (2.69, ±SD 1.12) and HCPs (2.45, ±SD 1.16), though with large variation. No statistical significant differences were found in importance, completeness, and user-friendliness between HCPs disciplines, nor between HCPs using the original ICP 1.0 or an adapted version.

### Qualitative

#### Importance

In the qualitative study, chapter 4 “Needs, wishes, and goals” was also identified as most important by parents and HCPs, because it serves as the foundation for all other chapters. Parents used the chapter to voice their needs and wishes in the care for their child. They wanted to describe who their child is, how to communicate with their child and how you could see how their child is doing, so that care could be aligned to the child’s specific needs. However, they felt this was not sufficiently represented in the ICP 1.0, and suggested this chapter to be expanded with more specific questions. Ideally, HCPs wish to explore the needs and wishes of child and parents in advance care planning conversations. HCPs would therefore like to see a more extensive version of chapter 4 that is more focused on advance care planning.

The overview of the care team including contact details, which is part of chapter 2 “General information,” was also mentioned as important by both respondent groups. It became apparent that this overview gave parents and HCPs insight into which HCPs were part of the child’s care team, which in turn supported interdisciplinary collaboration between HCPs across lines of care. Parents also found it helpful to have an overview of the contact details of all HCPs involved and to know who they could reach when necessary.

#### Completeness

Similar to the questionnaire, both parents and HCPs labeled the ICP overall as complete. However, at the same time they introduced points for improvement. HCPs missed having a chapter in which palliative sedation could be described. HCPs working in hospital felt the need to guide general practitioners and home care nurses in palliative sedation. Therefore, some HCPs have personalized the ICP 1.0 in their center by adding a step-by-step guide and medication plan for palliative sedation.

Primary care HCPs desired to add an overview of the resources and materials available at the child’s home, since it was not always clear where resources and materials originated from. Figuring this out was very time-consuming and could be avoided if everything was recorded.

Parents felt that end-of-life care could receive more extensive attention within the ICP. In addition, parents wanted to read more disease specific or palliative care-specific information in the ICP, for example, cessation of feeding during palliative sedation, to be able to understand what is happening. HCPs felt compelled to write down the orally given information so that parents could read it again at a later moment, but also to document disease-specific and end-of-life care information for other HCPs.

#### User-friendliness

Due to its completeness, HCPs evaluated the ICP 1.0 as very long and not user-friendly. This evoked fear that mistakes could be made in acute situations when they had to browse through the ICP and could not find relevant information quickly. They have a desire for a document with more manageable proportions that is easy to use. Parents and HCPs observed that the language in the ICP was often formal and/or medical and requested simpler language within the ICP. HCPs also perceived that the structure of the ICP was not always logical. For example, religion and spirituality are part of perception, while HCPs perceived these are separate topics and should have separated subheadings. The same applies to the social map and psychosocial aspects.

Parents and HCPs struggled with keeping the ICP up-to-date, especially the chapters 5 “Medication” and 7 “Symptomatology.” This was mainly due to the many changes needed in medication. HCPs were hesitant to update the ICP due to risk of errors. Making the ICP available in a digital environment was mentioned as a solution by parents and HCPs. By doing so, HCPs hoped to increase the visibility and thus awareness of changes made in the ICP.

In the interviews, chapter 7 “Symptomatology” was also often mentioned by HCPs as not user-friendly just like in the questionnaire. Although they found it important that the ICP displays all possible symptoms in the palliative phase to know what might be expected, these symptoms should be removable if they are not relevant for the individual child to keep the ICP organized.

## Discussion

This study reports the experiences of parents and HCPs with the content of the ICP 1.0 for PPC. Overall, the content of the ICP 1.0 was seen as important and complete by parents and HCPs, but at the same time additions were requested. The chapter describing the needs and wishes of child and parents was considered most important. Parents used this chapter to voice their needs and wishes for their child’s care and wished for this part to reflect better who their child is. HCPs would like to see this chapter expanded and to be more focused on advance care planning. HCPs mentioned to miss a chapter for palliative sedation, mainly to guide other HCPs. Parents and HCPs both wish to incorporate more information on the specific disease and on end-of-life care within the ICP. They also asked for simpler language use in the ICP. The ICP was appraised as very long and not user-friendly by both parents and HCPs, and according to them could improve by making the ICP available in a secure digital environment.

Parents and HCPs in our study both underpin the importance of tailoring care to the child and family’s needs. To be able to do so, HCPs emphasized the need to incorporate advance care planning conversation outcomes more effectively into the content of the ICP. Previous research has shown benefits of advance care planning, such as parallel planning, avoiding difficult conversations during crisis, and providing a document for parents to express their care preferences for their child.^18,19^ Since the development of the ICP, there have also been many developments in the field of advance care planning, leading to increased awareness among Dutch HCPs of the advantages of advance care planning in PPC.^20,21^ It is therefore important to see if advance care planning outcomes can be incorporated more effectively in the ICP.

Parents voiced the need for the content of the ICP to better represent who their child is as a person, how to communicate with their child and how you could see how their child is doing, so that good care could be provided. This is in line with previous studies that show that parents found it important that HCPs perceive their child as an individual rather than solely as a patient.^22,23^ When HCPs caring for the child are not aware of information about the child and agreements made regarding care and treatment, parents feel the need to remain hypervigilant to ensure continuity and quality of care.^22^ To prevent parents from feeling this need, it is important to study whether adjustments in the content of the Dutch ICP regarding information on the child are helpful.

Although the ICP was not meant as an informational document beyond the agreements on care and treatment of the child, both parents and HCPs wish to use the ICP to share information with parents and HCPs on the disease and on end-of-life care, including palliative sedation. Children in need of PPC have a wide range of underlying diseases, not uncommonly rare diseases.^24^ It is also relatively common in PPC to provide care for children with yet undiagnosed diseases.^25^ All those children have different needs. Parents know their child and the illness best. Furthermore, numerous HCPs are typically involved in the care for the child, but a consistent team is often lacking.^22^ It is therefore understandable that parents and HCPs feel the need to share information to ensure quality of care. Future research should look into whether an ICP is the right instrument to share this information.

It appears that the combination of describing the values and preferences of the child and parents, along with medical decisions and life-sustaining treatments, is essential for an ICP for PPC. However, as a result, the ICP is appraised as very long and not user-friendly. We believe the solution lies in making the ICP available in a secure digital environment. However, overcoming challenges, for example, linking software systems used across organizations, is imperative to make this feasibly.^26^ This requires subsequent research that can take years. Future research should therefore first prioritize the development of a comprehensive ICP, including all important content while at the same time having manageable proportions.

### Strengths and limitations

By using mixed methods and including parents and HCPs allowed us to study the user experiences regarding the content of ICP 1.0 from the perspective of all relevant actors involved and to build on the strengths of both questionnaires and interviews. It has allowed us to gain a comprehensive understanding of the essential content of an ICP, and to see what changes need to be made in the ICP to adhere to parents and HCPs wishes. This study also has some limitations. It was not possible to include children despite the efforts of the Children’s Palliative Care team contact persons to recruit children. They mainly could not be included due to developmental delays, communications issues, too young age or because they had already passed away. This is also the reality for including children in drawing up ICP’s. For this reason, parents of children receiving PPC are used to think and speak for their children and thereby able to share their experience with the ICP. The quantitative sample of parents is relatively small, however comprises of parents from both living and deceased children with a range of diagnosis thereby still providing a broad perspective on the content of the ICP. Having the interviews virtually with parents made it possible to obtain the interviews during the COVID-19 pandemic without risking contamination. However, an implication is that parents needed to have access to a digital device and internet to be able to participate. Nonetheless, online interviews have proven to be as effective as face-to-face interviews.^27^

## Conclusion

The content that is essential for an ICP in PPC as described by parents and HCPs is more extensive than previously described in literature. The ICP for PPC should include: medical details; care team overview including contact details; needs, wishes, and goals of child and parents guided by advance care planning conversation outcomes; treatment restrictions; religion and spirituality; social map; medication; symptom management; palliative sedation; and end-of-life care. To meet the needs of parents and HCPs considering importance and completeness of the content of the ICP and its user-friendliness, changes are necessary in the content of the ICP for PPC, and the ICP should preferably be made digitally available. Although various documents exist globally to facilitate anticipatory care or coordinating end-of-life care, it appears that the combination of describing the values and preferences of the child and parents, along with medical decisions and life-sustaining treatments, makes it a unique and comprehensive care plan.

## Supplemental Material

sj-docx-1-pcr-10.1177_26323524241277572 – Supplemental material for Parents’ and healthcare professionals’ experiences with the content of an individual care plan for pediatric palliative care: a mixed-method study Supplemental material, sj-docx-1-pcr-10.1177_26323524241277572 for Parents’ and healthcare professionals’ experiences with the content of an individual care plan for pediatric palliative care: a mixed-method study by Chantal Y. Joren, Marijke C. Kars, Leontien C. M. Kremer, Suzanne C. Hofman, Hester Rippen-Wagner, Ria Slingerland-Blom, Chantal van der Velden, Meggi Schuiling-Otten, A. A. Eduard Verhagen and Judith L. Aris-Meijer in Palliative Care and Social Practice

sj-docx-2-pcr-10.1177_26323524241277572 – Supplemental material for Parents’ and healthcare professionals’ experiences with the content of an individual care plan for pediatric palliative care: a mixed-method study Supplemental material, sj-docx-2-pcr-10.1177_26323524241277572 for Parents’ and healthcare professionals’ experiences with the content of an individual care plan for pediatric palliative care: a mixed-method study by Chantal Y. Joren, Marijke C. Kars, Leontien C. M. Kremer, Suzanne C. Hofman, Hester Rippen-Wagner, Ria Slingerland-Blom, Chantal van der Velden, Meggi Schuiling-Otten, A. A. Eduard Verhagen and Judith L. Aris-Meijer in Palliative Care and Social Practice

sj-docx-3-pcr-10.1177_26323524241277572 – Supplemental material for Parents’ and healthcare professionals’ experiences with the content of an individual care plan for pediatric palliative care: a mixed-method study Supplemental material, sj-docx-3-pcr-10.1177_26323524241277572 for Parents’ and healthcare professionals’ experiences with the content of an individual care plan for pediatric palliative care: a mixed-method study by Chantal Y. Joren, Marijke C. Kars, Leontien C. M. Kremer, Suzanne C. Hofman, Hester Rippen-Wagner, Ria Slingerland-Blom, Chantal van der Velden, Meggi Schuiling-Otten, A. A. Eduard Verhagen and Judith L. Aris-Meijer in Palliative Care and Social Practice

sj-pdf-4-pcr-10.1177_26323524241277572 – Supplemental material for Parents’ and healthcare professionals’ experiences with the content of an individual care plan for pediatric palliative care: a mixed-method study Supplemental material, sj-pdf-4-pcr-10.1177_26323524241277572 for Parents’ and healthcare professionals’ experiences with the content of an individual care plan for pediatric palliative care: a mixed-method study by Chantal Y. Joren, Marijke C. Kars, Leontien C. M. Kremer, Suzanne C. Hofman, Hester Rippen-Wagner, Ria Slingerland-Blom, Chantal van der Velden, Meggi Schuiling-Otten, A. A. Eduard Verhagen and Judith L. Aris-Meijer in Palliative Care and Social Practice

## Declarations

**Ethics approval and consent to participate:** For this study, we obtained approval from every medical ethics committee located at one of the seven University Hospitals (UMC Groningen reference number 201900822, UMC Utrecht, Radboud UMC, Maastricht UMC, Erasmus MC, Amsterdam UMC, Leids UMC) and Pediatric Oncology Centre (Princess Maxima Center). Study participants were fully informed of the study through the project website and informed consent was obtained digitally from all participants. All methods were performed in accordance with the relevant guidelines and regulations.

**Consent for publication:** Not applicable.

**Availability of data and materials:** The data generated or analyzed during this study are not publicly available due to the rare conditions that children have who qualify for PPC and therefore the possibility to be traced. We therefore want to protect their privacy by controlling access to the data. Data are available from the corresponding author on reasonable request and with permission from the principal investigator and coinvestigators.
