# Supplementary material for: Parents’ and healthcare professionals’ experiences with the content of an individual care plan for pediatric palliative care: a mixed-method study
Source: Palliat Care Soc Pract. 2024 Sep 18;18:26323524241277572. doi: 10.1177/26323524241277572 (PMC11418305; doi:10.1177/26323524241277572)
Supplement: sj-docx-1-pcr-10.1177_26323524241277572 – Supplemental material for Parents’ and healthcare professionals’ experiences with the content of an individual care plan for pediatric palliative care: a mixed-method study [file sj-docx-1-pcr-10.1177_26323524241277572.docx]

**Individual Care Plan Palliative Care for Children**

**1. Care plan information**

*This care plan*

- Is drawn up by: Name of the initial author

Name(s) of other author(s)

- Version number: Enter a number
- Is drawn up on: Select a date
- Approved by the child (always for 12+): Choose an item.

on: Select a date

- Approved by parents on: Select a date
- Approved by chief practitioner: Select a date
- To be revised on: Select a date ...

Description of what was discussed with parents/child (regarding prognosis, care goals etc.…, will follow later):
Has everything been discussed (including prognosis)? In what words? What does the child know?

**2. General information**

*Child’s information*

- Name Child’s name
- Date of birth Date of birth
- Address Street, House number
   Postal code, city
- Second address (if applicable) Name / description (e.g., mother’s address)
   Street, House number
   Postal code, city
- Phone numbers
  - Child Click here to enter text.
  - Father Click here to enter text.
  - Mother Click here to enter text.
- Email address Click here to enter text.
   Click here to enter text.
- Language spoken Choose item. …
- Proficiency in the Dutch language Yes
- Calculation details
  - Weight Click here to enter text. kg on Select date
  - Hight Click here to enter text. cm on Select date

*Medical situation*

- Diagnosis Click here to enter text.
- Date of diagnosis Select date
- Severity / prognosis:
  Click here to enter text.
- Relevant medical history:

Click here to enter text.

- Allergies Click here to enter text.

*Care team*

|  | | | |
| --- | --- | --- | --- |
| **24-hour Emergency number** | | Name / Number | |
|  | | | |
| **Chief practitioner** | | Name, title | |
| Phone | Phone number | Email address | Email address |
| After-hours | | Name / Number | |
|  | | | |
| **Care coordinator** | | Name, title | |
| Phone | Phone number | Email address | Email address |
| After-hours | | Name / Number | |
|  | | | |
| **Secondary practitioner /pediatrician** | | Name, title | |
| Phone | Phone number | Email address | Email address |
|  | | | |
| **Palliative care team coordinator** | | Name, title | |
| Phone | Phone number | Email address | Email address |
|  | | | |
| **General practitioner** | | Name, title | |
| Phone | Phone number | Email address | Email address |
| After-hours | | Name / Number | |
|  | | | |
| **Primary responsible nurse unit** | | Name, title | |
| Phone | Phone number | Email address | Email address |
|  | | | |
| **Home care** | | Name | |
| Primary contact person | | Name, title | |
| Phone | Phone number | Email address | Email address |
| Other contacts | | Name, title / Number | |
| After-hours | | Name / Number | |
|  | | | |
| **Pharmacy** | | Name | |
| Phone | Phone number | Email address | Email address |
| Afther-hours | | Name / Number | |
|  | | | |
| **Pediatrician reginal hospital** | | Name | |
| Phone | Phone number | Email address | Email address |
| After-hours | | Name / Number | |
|  | | | |
| **Psychologist** | | Name, institute | |
| Phone | Phone number | Email address | Email address |
|  | | | |
| **Medical social worker** | | Name, institute | |
| Phone | Phone number | Email address | Email address |
|  | | | |
| **Pedagogical Staff** | | Name, institute | |
| Phone | Phonen umber | Email address | Email address |
|  | | | |
| **Spiritual counselor** | | Name, institute | |
| Phone | Phone number | Email address | Email address |
|  | | | |
| **School / daycare** | | Name, institute | |
| Phone | Phone number | Email address | Email address |
|  | | | |
| **Other** | | Name, institute | |
| Phone | Phone number | Email address | Email address |
| **Other** | | Name, institute | |
| Phone | Phone number | Email address | Email address |
| **Other** | | Name, institute | |
| Phone | Phonen umber | Email address | Email address |

**3. Social map / psychosocial aspects**

*Data*

- *Family*
  - Father Name, age, accupation
  - Mother Name, age, occupation
  - Siblings Names and ages of siblings
- *Living situation*

Legal status, in case of separated partner(s), custody, and living arrangements

- *Housing conditions*

Practical aspects (e.g., ground floor residence, location of bedroom and bathrooms)

- *Medical facilities at home*

Description

- *Care support / Self-sufficiency*

Degree of independence / level of assistance required

- *Daily routine*

Description of daily routine

- *School / daycare*

Description of school/daycare, coordination with school/daycare

- *Leisure activities (sports / hobby)*

Brief description of sports/hobbies, names of associations, coordination

- *Social network of the child and family*

Grandparents, neighbors, friend

- *Other*

Other relevant aspects regarding the social map

*Perception*

- *Important aspects in the perception*

What are the family’s wishes/goals/important aspects of perception

- *Religion/spirituality*

What role does religion or spirituality play within this family?

- *Contact with psychosocial care providers*

Current contact with child psychologists

- *Relaxation exercises*

Interventions that have a positive effect on fear/anxiety of the child

- *Other*

Other aspects that are important with regard to perception

The manual contains extensive information on the psychosocial phases and aspects of palliative illness in the child, parents and environment.
*Psychosocial aspects that require attention are:*
Note here aspects as described in the manual that require special attention

*Other comments/details regarding social map/psychosocial aspects*E.g. relevant psychosocial history of family member

**4. Needs, wishes and goals**

*What are the child’s personal wishes, expectations, needs and (general) goals?*Free entry

*What are the personal wishes, expectations, needs and (general) goals of the parents?*Free entry

*Agreements made about care goals based on expectations and wishes*Free entry

*Any disputes/dilemmas*Note bottlenecks, for example where parents disagree with each other or with the clinician

*Have agreements been made regarding treatment restrictions?*

- Resuscitation policy Cleary record agreements
  - Circulation; drug support Choose an item. (explanation if needed)
  - Ventilation: suction Choose an item. (explanation if needed)
  - Ventilation: oxygen administration Choose an item. (explanation if needed)
  - Ventilation: mask and balloon Choose an item. (explanation if needed)
  - Ventilation: intubation/mechanical ventilation Choose an item. explanation if needed)
- Diagnostics Clearly record agreements
- Medication for infection (AB) Clearly record agreements
- Transfusion policy Clearly record agreements
- Admission Clearly record agreements
- Intensive care admission Clearly record agreements
- Fluid and nutrition Clearly record agreements
- Other: Clearly record agreements

*End of life*

Detail regarding end of life Agreements/wishes (e.g. location)
Possible autopsy/donation Discussed? Current wishes?

Farwell and funeral Current wishes? Discussed with child?
Bereavement care Agreements/wishes regarding bereavement care

**5. Medication including dosage**

*Daily/weekly*

Overview

Overview of medication

Ex.
XXX 3 daily xx mg
XXXX Once a week on Friday xx mg
XXXX 1 daily xx ug, iv.

Timetable

| Time | Name | Form | Dose |
| --- | --- | --- | --- |
| … | ... | … | … |

*If needed, currently:*Description of medication

*Options to add, in case of…*Descripton of medication and when to give it

*Present at the child/parents’ home*
What is available in the house? (think of future medication!) Both medication and material.

**6. Nutrition**

*Wishes of parent/child regarding (tube)feeding* : Click here if you want to enter text.

*Description of feeding pattern*Description of daily nutrition/fluid intake/tube feeding)

*Agreements regarding fluids, nutrition and supplements*
Write down clearly

**7. Symptomatology**

Indicate which symptoms are currently relevant and which symptoms can be expected.

**Symptoms - Current**

Click here if you want to enter text.

**Symptoms – Take into account possibly expected during diagnosis/prognosis**

Click here if you want to enter text.

**Symptoms – General wishes of parents/child**

Click here if you want to enter text.

**Symptoms – General comments**

Click here if you want to enter text.

------------------------------
**7a. Pain**

**Current situation:**  Click here if you want to enter text.

**Expectation / “Is it likely that this will occur in this palliative process?”:**Click here if you want to enter text.

**Goals child/parents:** Click here if you want to enter text.

Specific description of current situation: Pain scores, localizations, course

*Diagnostics*

Measuring instrument pain: Make your choice ...

*Treatment - Medicinal*Nociceptive pain

**! Currently in step: 0 , any additions/adjustments:** Click here if you want to enter text. **!**

Step 1 – not opioids

*Step 1a - Paracetamol (*[*KF*](https://www.kinderformularium.nl/geneesmiddel/21/paracetamol)*)*

Dosage: Paracetamol Choose an item., Choose an item dd ... mg.

Administration form: e.g tablet

*Step 1b – NSAIDs (*[*KF ibu*](https://www.kinderformularium.nl/geneesmiddel/18/ibuprofen)*,* [*KF diclo*](https://www.kinderformularium.nl/geneesmiddel/17/diclofenac)*)*

Dosage: Choose an item. Choose an item., Choose an item. dd ... mg.

Step 2 – Opioids for mild pain

*Tramadol (*[*KF*](https://www.kinderformularium.nl/geneesmiddel/12/tramadol)*)*

Dosage: Step 1. Tramadol Choose an item., Choose an item. dd ... mg.
 Step 2. Tramadol Choose an item., Choose an item. dd ... mg.

Step 3 – Opioids for severe pain

NB. **Avoid codeine, consider oxycodone, buprenorphine and PCA pump**

*Morphine (*[*KF*](https://www.kinderformularium.nl/geneesmiddel/16/morfine)*)*

Dosage:

Short-acting morphine Choose an item., Choose an item. ... mcg Kies een Choose an item.

Long-acting morphine Choose an item., Choose an item. ... mcg Choose an item..
Fentanyl patch: Click here if you want to enter text.

Rule for breakthrough pain: Choose an item.. ...

Schedule adjustment for long-acting opiate: Choose an item.. ...

*Oxycodon (*[*KF*](https://www.kinderformularium.nl/geneesmiddel/603/oxycodon)*) of buprenorfine (*[*FK*](https://www.farmacotherapeutischkompas.nl/preparaatteksten/b/buprenorfine.asp)*)*

Dosage: Choose an item. Choose an item., Choose an item. dd ... mg.

Neuropathic pain

NB. **Avoid phenytoin, carbamazepine and valproate, consider opioids**

Tricyclic antidepressant ([FK](https://www.farmacotherapeutischkompas.nl/preparaatteksten/a/amitriptyline%20(verwijzing).asp))

Dosage: Amitriptyline Choose an item., Choose an item. dd ... mg.

*Depending on the effect and side effects, gradually increase every 3-7 days if no effect yet.*

Antiepileptic drugs ([FK](https://www.farmacotherapeutischkompas.nl/preparaatteksten/g/gabapentine%20(verwijzing).asp))

Dosage: Choose an item. Choose an item., Choose an item. dd ... mg.

Other pain (e.g. bone pain)

Other pain medication: Click here if you want to enter text.

*Treatment – Non-medicinal*Actions taken: Click here if you want to enter text.

------------------------------
**7b. Nausea/vomiting**

**Current situation:**  Click here if you want to enter text.

**Expectation / “Is it likely that this will occur in this palliative process?”:**Click here if you want to enter text.

**Goals child/parents:** Click here if you want to enter text.

*Diagnostics*Keeping diary frequency/duration: Choose an item ...

*Treatment – Non-medicinal*Actions taken: Click here if you want to enter text.

*Treatment - Medicinal*

Currently in step: 0 , any additions/adjustments: Click here if you want to enter text.

Step 1

*Stap 1a - 5-HT3-receptor antagonist (*[*KF ond*](https://www.kinderformularium.nl/geneesmiddel/30/ondansetron)*)*

Dosage: Ondansetron Choose an item., Choose an item. dd … mg.

*Stap 1b – D2-receptor antagonist (*[*KF domp*](https://www.kinderformularium.nl/geneesmiddel/27/domperidon)*)*

Dosage: Domperidon Choose an item., Choose an item. dd … mg.

Further medication for nausea/vomiting (see manual)

Click here if you want to enter text.

------------------------------

**7c. Constipation**

**Current situation:**  Click here if you want to enter text.

**Expectation / “Is it likely that this will occur in this palliative process?”:**Click here if you want to enter text.

**Goals child/parents:** Click here if you want to enter text.

*Treatment- Non-medicinal*Actions taken: Click here if you want to enter text.

*Treatment - Medicinal*Medication and dosage: Click here if you want to enter text.

------------------------------

**7d. Dyspnea**

**Current situation:**  Click here if you want to enter text.

**Expectation / “Is it likely that this will occur in this palliative process?”:**Click here if you want to enter text.

**Goals child/parents:** Click here if you want to enter text.

*Diagnostics*

Measuring instrument dyspnea: Choose an item. ...

*Treatment – Non-medicinal*Measures taken: E.g. in case of shortness of breath )2 for comfort, posture advice, etc.

*Treatment - Medicinal*

Dyspnea - *Morphine (*[*KF*](https://www.kinderformularium.nl/geneesmiddel/16/morfine)*)*

Dosage: Step 1. Morphine Choose an item., Choose an item. ... mcg Choose an item..
 Step 2. Morphine Choose an item., Choose an item. ... mcg Choose an item..

Next steps: Click here if you want to enter text.

Restlessness/anxiety in dyspnea - *Benzodiazepines (*[*KF lora*](https://www.kinderformularium.nl/geneesmiddel/139/lorazepam)*,* [*KF mida*](https://www.kinderformularium.nl/geneesmiddel/140/midazolam)*), as an addition to morphine*

Dosage: Choose an item. Choose an item., Choose an item. dd ... mg.

Other medication for dyspnea: Click here if you want to enter text.

------------------------------

**7e-1. Cough**

**Current situation:**  Click here if you want to enter text.

**Expectation / “Is it likely that this will occur in this palliative process?”:**Click here if you want to enter text.

**Goals child/parents:** Click here if you want to enter text.

*Comments on current situation*Click here if you want to enter text.

*Treatment - Cause*Actions taken: Click here if you want to enter text.

*Treatment- Non-medicinal*

Actions taken: Click here if you want to enter text.

*Treatment - Medicinal*Medication and dosage: Click here if you want to enter text.

------------------------------

**7e-2. Rattling**

**Current situation:**  Click here if you want to enter text.

**Expectation / “Is it likely that this will occur in this palliative process?”:**Click here if you want to enter text.

**Goals child/parents:** Click here if you want to enter text.

*Comments on current situation*Click here if you want to enter text.

*Treatment – Non-medicinal*Actions taken: Click here if you want to enter text.

*Treatment - Medicinal*Medication and dosage: Click here if you want to enter text.

------------------------------

**7f. Fatigue**

**Current situation:**  Click here if you want to enter text.

**Expectation / “Is it likely that this will occur in this palliative process?”:**Click here if you want to enter text.

**Goals child/parents:** Click here if you want to enter text.

*Comments on current situation*Click here if you want to enter text.

*Diagnostics*Ever go through screening list? Choose an item. – Name screenings list

*Treatment – Non-medicinal*Actions taken: Click here if you want to enter text.

*Treatment - Medicinal*Medication and dosage: Click here if you want to enter text.

------------------------------

**7g. Anxiety and depression**

**Current situation:**  Click here if you want to enter text.

**Expectation / “Is it likely that this will occur in this palliative process?”:**Click here if you want to enter text.

**Goals child/parents:** Click here if you want to enter text.

*Comments on current situation*Click here if you want to enter text.

*Diagnostics*Ever go through screening list? Choose an item. – Name screenings list
Possible other causes/triggers: Choose an item. - Click here if you want to enter text.

*Treatment – Non-medicinal*Actions taken: Click here if you want to enter text.

*Treatment – Medicinal*Medication and dosage: Click here if you want to enter text.

 ------------------------------
**7h. Neurological symptoms**
**7h-1. Epilepsy**

**Current situation:**  Click here if you want to enter text.

**Expectation / “Is it likely that this will occur in this palliative process?”:**Click here if you want to enter text.

**Goals child/parents:** Click here if you want to enter text.

*Comments on current situation*Click here if you want to enter text.

*Treatment – Medicinal*

- Attack medication (Attack duration of 5 minutes is t=0 for medicinal interventions)
- Step 1, t=0, give Choose an item. Choose an item …mg one-off

Step 2, t=5, give Choose an item. Choose an item. …mg one-off

Insertion of IV desired by child/parents? Choose an item., possibly bone needle? Choose

- Step 3, t=10, intravenous Choose an item. …mg
- Maintenance medication: Click here if you want to enter text.

**7h-2. Movement disorders, spasticity, disability symptoms**

**Current situation:**  Click here if you want to enter text.

**Expectation / “Is it likely that this will occur in this palliative process?”:**Click here if you want to enter text.

**Goals child/parents:** Click here if you want to enter text.

*Comments on current situation*Click here if you want to enter text.

*Treatment – Non-medicinal*Actions taken: Click here if you want to enter text.

*Treatment - Medicinal*Medication and dosage: Click here if you want to enter text.

 ------------------------------
**7i. Hematological phenomena**
**Current situation:**  Click here if you want to enter text.

**Expectation / “Is it likely that this will occur in this palliative process?”:**Click here if you want to enter text.

**Goals child/parents:** Click here if you want to enter text.

*Diagnostics*Last Hb: value mmol/l op date.
 Trombo: value x 10^9^/l op date.
other diagnostics: Click here if you want to enter text.

*Treatment- Non-medicinal*

History of transfusions: Describe previous transfusions + effect.
History of bleeding: Describe previous bleeding + measures taken + effect.
Emergency kit acute serious life-threating bleeding at home: Choose an item. …
Actions taken: Click here if you want to enter text.

*Treatment - Medicinal*Medication and dosage: Click here if you want to enter text.

 ------------------------------
**7j. Skin phenomena**

**7j-1. Itch**

**Current situation:**  Click here if you want to enter text.

**Expectation / “Is it likely that this will occur in this palliative process?”:**Click here if you want to enter text.

**Goals child/parents:** Click here if you want to enter text.

*Comments on current situation*Click here if you want to enter text.

*Treatment – Non-medicinal*Actions taken: Click here if you want to enter text.

*Treatment - Medicinal (Both local and systemic)*Medication and dosage: Clear description of ointments, regularity, quantity, spots. etc.

 ------------------------------
**7j-2. Wounds/decubitus/mucositis**

**Current situation:**  Click here if you want to enter text.

**Expectation / “Is it likely that this will occur in this palliative process?”:**Click here if you want to enter text.

**Goals child/parents:** Click here if you want to enter text.

*Comments on current situation*Click here if you want to enter text.

*Diagnostics*Extensive description per location; redness, size, depth and shape, wound edges, maceration, the degree of exudate and bleeding, tendency, odor, swelling, heat and pain. Gradation (stage) per spot!

*Treatment*Actions taken: Click here if you want to enter text.

 ------------------------------
**7k. Other**

Click here if you want to enter text.

**8. Alternative therapies and relaxation/wellness**

Overview complementary therapies
Click here if you want to enter text.

Relaxation/wellness actions
Click here if you want to enter text.

**9. History of change**

Last updated: Choose date

Main changes: Click here if you want to enter text.

**10. Other**

Click here if you want to enter text.
