# Supplementary material for: Parents’ and healthcare professionals’ experiences with the content of an individual care plan for pediatric palliative care: a mixed-method study
Source: Palliat Care Soc Pract. 2024 Sep 18;18:26323524241277572. doi: 10.1177/26323524241277572 (PMC11418305; doi:10.1177/26323524241277572)
Supplement: sj-docx-2-pcr-10.1177_26323524241277572 – Supplemental material for Parents’ and healthcare professionals’ experiences with the content of an individual care plan for pediatric palliative care: a mixed-method study [file sj-docx-2-pcr-10.1177_26323524241277572.docx]

**Topic guide parents**

Introduction to the research:

- Thanks for participating. Both questionnaire and interview.
- Short explanation about research; cause, aim, phasing.
- Interview lasts 45-60 minutes, will be recorded. Afterwards written down anonymously, video/audio files are deleted.
- If there are any ambiguities after transcribing, or if unclear what was meant when reading back, I will contact you.
- No right or wrong answers, is about how it’s been for you
- Any questions?
- Agree we start? Indicate when a break is needed.

Questionnaire focused mainly on lay-out and completeness. This conversation is more about what the ICP has meant in the care for your child, in the collaboration with and between healthcare professionals.

Opening question: tell me about [child].

Would you please go back to the moment when the ICP was first discussed and outline that situation from there until the moment the ICP was finalized; when wat this ins year/disease process, who took the initiative, what was the reason, how did the process proceed.

| Theme | Interview question | Any additional questions |
| --- | --- | --- |
| Process | - Back to the moment when the ICP was first discussed – outline the situation from there to the moment the ICP was finalized | - Year - At what stage in the illness trajectory? - Was that a logical moment for you? - Who took the initiative? - What was the reason? - What was the goal? - One conversation or multiple? - How did you feel when the ICP was discussed? - How were those conversations for you at that moment? - How do you look back on it now? - Which healthcare professionals were involved? |
| Input parent/child | - Can you describe your role in completing the ICP? - In what way has consideration been given to what is important for your child and family? | - Have you been involved in the drawing up? - Was the child involved in the drawing up? - How do you look back on the involvement of you/your child? - How is this reflected in the ICP? - How were choices/decisions made? - Does the child do something with the ICP themselves? |
| Use of the ICP | - How do healthcare professionals deal with the ICP? - What do you think of the content of the ICP? - Which part of the ICP do you attach value to? | - In thinking about the future, do they recognize the child and the situation in it - Feasibility - Extent/manner of use - Do you have the impression that it has already been looked at before you enter the conversation? - Situation in which the ICP was not used while you expected it to be - Are there subjects missing? If so, which ones? - Are there parts that do not apply to your child? If so, which parts? |
| Meaning ICP in daily life | - Could you describe a situation in which the ICP played a role? - What does the ICP mean to you as a parent? - What does the ICP mean for your family? | - How was the ICP perceived by them? - What did it do? - Who did you discuss it with? - Is it present at your home? - Do you ever look into it? - What do you think about it then? - When do you look at it? |
