# Supplementary material for: Parents’ and healthcare professionals’ experiences with the content of an individual care plan for pediatric palliative care: a mixed-method study
Source: Palliat Care Soc Pract. 2024 Sep 18;18:26323524241277572. doi: 10.1177/26323524241277572 (PMC11418305; doi:10.1177/26323524241277572)
Supplement: sj-docx-3-pcr-10.1177_26323524241277572 – Supplemental material for Parents’ and healthcare professionals’ experiences with the content of an individual care plan for pediatric palliative care: a mixed-method study [file sj-docx-3-pcr-10.1177_26323524241277572.docx]

**Topic guide drawing up ICP**

- Opening
- Introduction research
- Practical aspects interview

**Strengths and weaknesses (20 minutes)**

- Which aspects of the ICP need significant improvement, and why?
- Which aspects of the ICP should not be changed, and why?

Use MURAL. Consider aspects such as form, content, development process, purpose of the ICP, etc. The entirety of the ICP.

After MURAL (10 minutes). Discuss in the group. Take turn, giving everyone a chance to speak.

- Which of the comments made do you find the most important?
- Which ones cause you the most trouble?

Additional questions:

- Is something missing? If so, what?
- What can be removed?
- What annoys you?
- Does it affect your use of the ICP?

**Current situation (40 minutes)**

Depending on the dynamics, possibly use a set of statements about the ICP. A green object in front of the camera for agreement, a red object for disagreement. For groups that have already delved into pros and cons, move on to the opening question.

Drawing up of the ICP
Statements
- The ICP should be drawn up by the chief practitioner
- The ICP is drawn up within two months after diagnosis
- ACP tools are indispensable in the development of an ICP

Opening Question

You all have experience with drawing up ICPs. I would like you to think back to a situation where the idea of an ICP first cam to your mind until it became finalized, and take me through that process. Who took the initiative, how did you bring it up, who were involved, how long did it take, at what point in the illness trajectory, and why did you do it at that moment, etc.? Whoever want to speak, please share.

Additional questions:

- How does the process go?
  - At what point in the illness trajectory do you take the initiative? And why at that point?
  - How do you bring it up?
  - What was the reason behind it?
  - What was the goal?
  - How did the process unfold from the initial initiative to the final ICP?
  - How was this process for you?
- For whom and why?
  - Should every child in palliative care have an ICP? Why or why not?
  - When do you use it or not?
  - When do you implement it?
  - Why is it so challenging to discuss it early in the trajectory?
  - Who is responsible for the ICP? Is it the case currently?
- Family-centered
  - How is it ensured that the needs of the child and family are taken into account?
  - How is this reflected in the ICP?
  - Who do you work with- both parents or the more approachable parent?

**Function of the ICP in healthcare (20 minutes)**

Possible statements

- So far, the ICP has always played a significant role in the care for the child and the family.
- The ICP is a living document for all involved parties.
- Adjustments in care can be made by all involved healthcare professionals in the ICP.
- The ICP should be a standard part of care plans in primary healthcare.

Further explore points raised in the first part.
Additional questions:

- What does the ICP add?
- What is the significance of an ICP in the palliative trajectory?
- What is its purpose?
- How is it handled by your direct colleagues?
- How do you use the ICP? (i.e., how do you work with it?)
- Does the ICP facilitate collaboration between parents/healthcare professionals and among different lines of care??

**Ideal situation (5-10 minutes)**

After discussing the current situation, we’ll now delve into how you think the ICP should ideally be. Using the same Mural link, you will see two sections: “Dreams” and “Other”. If anything is possible, how would the ideal ICP look like? Lay-out, content, process. How would you prefer it to be? Use the left section for your response and the right section for additional comments or suggestions. Use the same color of the post-it notes as before (a quick reminder), or add your name in the text box. We won’t go into extensive questioning, only if there are any uncertainties.

**Closing (5-10 minutes)**

- Brief summary of the discussed points
- Is there anything that hasn't been asked or addressed?
- In one sentence, reflect on the focus group meeting – how was it for you?
- Note down who would like to receive the result/newsletter and refer them to the website.
- Thank everyone for their participation.

**Topic guide working with the ICP**

- Opening
- Introduction research
- Practical aspects interview

**Strengths and weaknesses (20 minutes)**

- Which aspects of the ICP need significant improvement, and why?
- Which aspects of the ICP should not be changed, and why?

Use MURAL. Consider aspects such as form, content, development process, purpose of the ICP, etc. The entirety of the ICP.

After MURAL (10 minutes). Discuss in the group. Take turn, giving everyone a chance to speak.

- Which of the comments made do you find the most important?
- Which ones cause you the most trouble?

Additional questions:

- Is something missing? If so, what?
- What can be removed?
- What annoys you?
- Does it affect your use of the ICP?

**Current situation (40 minutes)**

Depending on the dynamics, possibly use a set of statements about the ICP. A green object in front of the camera for agreement, a red object for disagreement. For groups that have already delved into pros and cons, move on to the opening question.

**Working with the ICP**

Possible statements

- So far, the ICP has always played a significant role in the care for the child and the family.
- The ICP is a living document for all involved parties.
- Adjustments in care can be made by all involved healthcare professionals in the ICP.
- The ICP should be a standard part of care plans in primary healthcare.

Opening question

You all have experience with the ICP, and I would like to ask you to think back to one of the children for whom the ICP played a significant role in care and take me through what it looked like. What did you do with the ICP? What was its added value? What role did the ICP play in collaboration with other healthcare professionals? Who is responsible for it? What were the obstacles? Who made adjustments, etc.?

Additional questions:

- Value in practice
  - How is it to work with an ICP?
  - What does the ICP add? For parents and for you?
  - How is it handled? Do you always know if there is an ICP? Can you access it? Who informed you about it?
  - What is the function of the ICP in your work?
  - What is the significance of an ICP in daily life?
  - How is it among your direct colleagues? Does everyone use it?
- Process
  - Does the ICP help in collaboration between parents/healthcare professionals and among different lines of care?
  - Who should create the ICP?
  - Who is allowed to make adjustments?
  - How do you signal changes?
  - What role do ACP tools play in this?
  - When do you belong in the inner circle with access to the ICP?
- Content
  - What do you think about it?
  - Do you find it helpful?
  - Do you see the child and family reflected in it?
- Combination with other care plans
  - How does the ICP align with other care plans?
  - What are the similarities/differences? Can they be integrated, or do the serve different purposes?
  - How much attention to palliative care is there in the other care plans?
  - How is that determined?

**Ideal situation (5-10 minutes)**

After discussing the current situation, we’ll now delve into how you think the ICP should ideally be. Using the same Mural link, you will see two sections: “Dreams” and “Other”. If anything is possible, how would the ideal ICP look like? Lay-out, content, process. How would you prefer it to be? Use the left section for your response and the right section for additional comments or suggestions. Use the same color of the post-it notes as before (a quick reminder), or add your name in the text box. We won’t go into extensive questioning, only if there are any uncertainties.

**Closing (5-10 minutes)**

- Brief summary of the discussed points
- Is there anything that hasn't been asked or addressed?
- In one sentence, reflect on the focus group meeting – how was it for you?
- Note down who would like to receive the result/newsletter and refer them to the website.
- Thank everyone for their participation.
